# The Comparison of the Effects of Acute and Repeated Morphine Administration on Fast Synaptic Transmission in Magnocellular Neurons of Supraoptic Nucleus, Plasma Vasopressin Levels, and Urine Volume of Male Rats 

**Published:** 2014

**Authors:** Mitra Yousefpour, Nima Naderi, Zahra Mansouri, Mahyar Janahmadi, Amir-Mohammad Alizadeh, Fereshteh Motamedi

**Affiliations:** a *Neuroscience Research Center, Shahid Beheshti University of Medical Sciences.*; b ^b^*Department of Physiology, Faculty of Medicine, Shahid Beheshti University of Medical Sciences. *; c *Department of Physiology, Faculty of Medicine, Army University of Medical Sciences. *; d *Department of Pharmacology and Toxicology, Faculty of Pharmacy, Shahid Beheshti University of Medical Sciences. *

**Keywords:** Supraoptic nucleus, Morphine, Arginine vasopressin, Urine volume, sIPSC, sEPSC

## Abstract

The activity of the magnocellular neurons (MCNs) of supraoptic nucleus (SON) is regulated by a variety of excitatory and inhibitory inputs. Opioids are one of the important compounds that affect these inputs at SON synapses. In this study, whole-cell patch clamp recording of SON neurons was used to investigate the effect of acute and repeated morphine administration on spontaneous inhibitory and excitatory post synaptic currents (sIPSCs and sEPSCs) in MCNs. While acute bath application of morphine to brain slice of intact rat produced an increase in sEPSCs frequency and a decrease in sIPSCs frequency, repeated *in-vivo *administration of morphine produced opposite effect. Moreover, repetitive i.c.v. administration of morphine for three consecutive days caused significant increase in urine volume, but had no significant alteration in water consumption compared to control group. The increase in urine volume was consistent with a significant decrease in plasma arginine vasopressin (AVP) levels after repetitive *i.p*. morphine administration. The results suggest that acute administration of morphine stimulates whereas repeated administration of morphine inhibits the MCNs. Morphine-induced MCN inhibition could result in diminished plasma AVP levels and eventually an increase in urine volume of rats.

## Introduction

The activity of magnocellular neurons (MCNs) is regulated by both intrinsic conductance and a variety of excitatory/inhibitory inputs. The major excitatory afferents in the hypothalamus are glutamatergic (1, 2) and inhibitory afferents are largely GABAergic (2, 3). There is a wide variety of transmitters which can modulate the efficacy of afferent transmission into these cells (4, 5). Opioids are amongst important transmitters that have been shown to influence MCNs neuronal activity (6-8). 

A number of studies indicate that opioids may be involved in controlling release of Oxytocin (OXT) and Arginine-vasopressin (AVP). The supraoptic nucleus (SON) receives opioid innervations from fibers originating from other brain regions and contains high levels of μ and κ opioid receptor binding sites (9). Opioid compounds produce different effects on electrical activity of MCNs (10). While in a previous study acute morphine administration inhibited oxytocin neuron firing rate (11), other studies showed that acute morphine administration produced excitatory effects on SON neurons which is possibly dose dependent (10, 12). Also, it was shown that acute administration of morphine induces FOS immune reactivity in SON but repeated administration of morphine attenuates FOS production (13). Similar to morphine effects on SON neurons, controversial effects of morphine on plasma AVP levels were also reported. Some studies showed that morphine stimulates AVP secretion (14-16) while others report no significant increase in plasma AVP level (17, 18). In addition, AVP was elevated in the SON after acute morphine injection but the AVP content was decreased in repeated administration of morphine (19).

The cell bodies of most of neurons projecting to the posterior pituitary lie in the SON, rather than paraventricular nucleus (20). Thus SON represents a potentially more important site at which opiates might influence the hypothalamic control of neurohypophysial hormone release. Since morphine-induced control of neurosecretion might be due to the modulation of synaptic inputs, we performed the present study to investigate the effects of acute and repeated morphine administration on spontaneous inhibitory and excitatory post synaptic currents (sIPSCs and sEPSCs) in MCNs of SON. We also investigated changes in plasma AVP concentration as well as urine volume in response to morphine administration in male rats.

## Experimental

*Materials and methods*

All experiments in the present study were conducted in accordance with National Institute of Health Guide for the Care and Use of Laboratory Animals (NIH Publications No. 80-23) revised 1996. Male Wistar rats (70-100 g, 3–4 weeks old) were purchased from Pasteur institute (Tehran, Iran). Animals were kept in a room with 12 h/12 h light/dark cycle (lights on at 0700) and controlled temperature (22 ± 2 °C) before conducting experiments.

*Chemicals*

Drugs used in this study were the opioid receptor agonist morphine sulfate (Temad, Tehran, Iran), the opioid receptor antagonist naloxone (Sigma, St. Louis, MO, USA), the GABA_A _receptor antagonist bicuculline (Fluka, Switzerland), the competitive NMDA receptor antagonist D-(-)-2-Amino-5-phosphonopentanoic acid (D-AP5; Sigma, St. Louis, MO, USA) and the non-NMDA receptor antagonist 6-Cyano-7-nitroquinoxaline-2,3-dione (CNQX; Ascent scientific, UK). 

*Electrophysiological recording from SON neurons*

*Slice preparation*

Animals were anesthetized with ether and decapitated. The brain was quickly removed and placed in ice-cold (0-2 °C) slicing solution contained (in mM) 87 NaCl, 2.5 KCl, 1.25 NaH_2_PO_4_, 7 MgCl_2_, 0.5 CaCl_2_, 25 NaHCO_3_, 25 Glucose and 75 Sucrose, saturated with 95% O_2_ and 5% CO_2_. Coronal slices (250 μm) were cut with a vibratome (Campden instruments Co. UK) from a block of tissue containing the hypothalamus. Slices including the SON were hemisected along the midline and allowed to recover for at least 1 h in 32-34 °C. The slice was then transferred into a recording chamber in which it was submerged and continuously perfused with artificial cerebrospinal fluid (ACSF) (0.5 mL/min). The composition of the ACSF was as follows (in mM): 126 NaCl, 2.5 KCl, 1.2 Na_2_HPO_4_, 18 NaHCO_3_, 1.2 MgCl_2_, 2.4 CaCl_2_, 11 glucose; pH 7.4 (295 mOsm/Kg).

*Drug application for patch-clamp recording*

For acute morphine administration, the drug was bath applied. Minimum effective concentration of morphine (25 μM) was selected based on preliminary experiments. Greater concentrations did not produce significantly more effective effects. Appropriate stock solutions were made and diluted with ACSF just before application. Drug applications were performed in a constant flow rate of 0.5 mL/min for a period of 10 min and then plain ACSF was substituted during the rest of recording. In order for repeated morphine administration, *i.p*. injection of the appropriate doses of morphine (10, 20, 30, and 50 mg/Kg/day) for 4 consecutive days was performed. In last day, the presence of withdrawal symptoms following naloxone administration was used as confirmation of the development of tolerance and dependence.

*Whole cell Patch-clamp recording*

To characterize the rapid membrane effects of morphine on MCNs, we performed whole cell patch clamp recordings in neurons of the SON in acutely prepared hypothalamic slices. MCNs were identified visually by their relatively large somatic size and position in the SON using infrared differential interference contrast (IR-DIC) microscopy (BX51WI Olympus, Tokyo, Japan). Patch-clamp recording pipettes (3–7 MΩ) were filled with a solution containing the following (in mM): 130 CsCl (for IPSC) or 130 potassium gluconate (for EPSC), 10 HEPES, 1 CaCl_2_, 1 MgCl_2_, 5 EGTA, 1 NaCl, 2 Na_2_-ATP and pH adjusted to 7.2 with CsOH (for IPSC) or KOH (for EPSC). The cells were recorded at 32 ± 2 °C. Spontaneous EPSCs were recorded as inward synaptic currents at a holding potential of -70 mV in presence of the GABA_A_ receptor antagonist bicuculline (30 μM) and were blocked by the ionotropic glutamate receptor antagonists AP-5 (50 μM) and CNQX (20 μM). Spontaneous IPSCs were recorded with cesium-containing electrodes as outward synaptic currents at a holding potential of 0 mV in presence of the AP-5 (50 μM) and CNQX (20 μM) and were blocked by the GABA_A_ receptor antagonist bicuculline (30 μM). Data were collected only after a 15-20 min baseline recording during which a stable amplitude and frequency of synaptic currents were observed. For each cell, an epoch of 5 min immediately before drug administration was considered as control values and the rest of recording was compared with this pre-treatment control values. Membrane currents were recorded using an amplifier (Axopatch 200B, Molecular device, USA), low-pass filtered at 2 kHz, and digitized using the Digidata 1322A (Axon instrument, USA). Series resistance (up to 20 MΩ) was monitored online during the recording and cells were excluded from data analysis if more than 15% change occurred during the course of the experiment. No whole cell series resistance compensation was made during recording of spontaneous events. Spontaneous events were detected with the threshold levels of 3-times the baseline noise using Mini Analysis Program (Synaptosoft Inc., NJ, USA). The amplitude of the synaptic current was calculated from the baseline to the peak of each response. 

*Measurement of daily water consumption and urine volume of rats*

In order to eliminate the peripheral effects of morphine on urine volume and water consumption (*e.g*. effects on kidneys), the drug was administered into the lateral ventricle of rat brain. The animals were anesthetized with *i.p*. injection of ketamine (85 mg/Kg) and xylazine (15 mg/Kg). Then rats were placed in stereotaxic apparatus (Stoelting, USA) and implanted with guide cannula (8mm, 23-gauge) aimed at a site 1 mm above the right lateral ventricle according to following coordinates: 1 mm posterior and 1.6 mm lateral to the bregma at a depth of 3.5 mm from the skull surface (21). Two jeweler screws were inserted into the skull and the cannula was fixed using dental cement. Then the cannula was closed with a stylet. After surgery, the animals were allowed a week to recover in their home-cage. For the intracerebroventricular (i.c.v.) administration of morphine, animals were gently hand-restrained and drug infusions were made using an injection needle (30-gauge) inserted into the guide cannula connected through a polyethylene (PE-20) tube to a 25 μL Hamilton syringe. Drug infusions were performed with infusion rate of 0.25 mL/min using an infusion pump (model NE-1000, New Era Pump Systems Inc., USA). Rats received daily i.c.v. administration (at 9:00 a.m.) of different doses of morphine (20, 100, and 200 μg/rat) or saline for three consecutive days and were kept in methabolic cages (Borj sanat, Tehran, Iran). During this period, animals had free access to food and water. Water consumption and urine volume were measured daily prior to drug administration (at 8:00 a.m.). 

*Measurement of plasma AVP levels*

Rats were treated by i.c.v. administration of morphine (100 μg/rat; selected based on preliminary studies) for three consecutive days as described above. On day 3, 45 min after drug administration, rats were anesthetized by ether and decapitated. The blood samples were collected in microtubes containing EDTA, aprotinin, and PMSF (for prevention of coagulation and proteases activity) and centrifuged for 10 min in 3500 g at 4 ºC. The plasma was then separated and stored at -80 ºC prior to assay. Plasma AVP levels was measured using commercially available kits (USCN Life Science & Technology, Wuhan, China) by ELISA technique. The delay time between last drug administration and blood sample collection was selected according to the previous results of our lab (22).

*Data analysis*

Data were presented as mean ± SEM (standard error of mean) and were analyzed by Prism^®^ 5 (GraphPad Software Inc., 2007). Paired t-test, one-way or two-way ANOVA followed by Dunnett’s or Bonferroni’s post tests were used as appropriate. A p-value less than 0.05 were considered to be statistically significant.

## Results

Spontaneous synaptic currents were recorded from supraoptic neurons. Both sEPSCs and sIPSCs were observed without any stimulus.

*Different actions of acute and chronic morphine administration*

**Figure 1:**
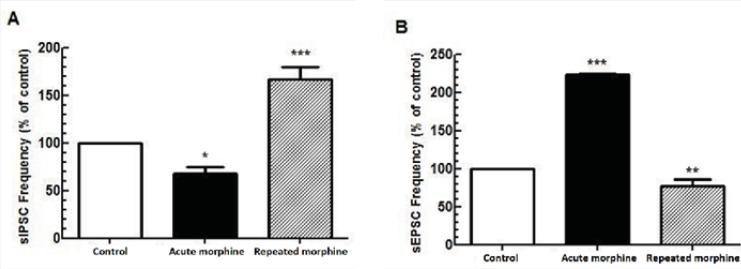
Comparison between the effect of acute and repeated morphine administration on MCNs activity. (A) Bath application of morphine (25 μM) elicited reduction in the frequency of sIPSC (n=7) but repeated *in-vivo *administration of morphine increased the frequency of sIPSC (n=6). (B) Bath application of morphine (25 μM) increased the frequency of sEPSC (n=5) whereas repeated morphine administration decreased the frequency of sEPSC (n=5). Each bar represents mean ± SEM.

**Figure 2:**
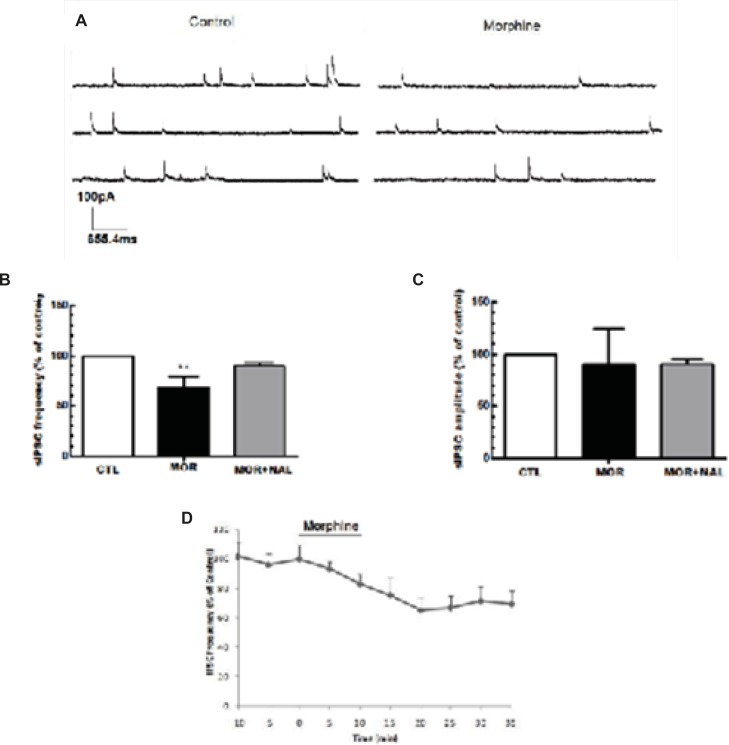
Acute morphine-induced suppression of GABA release onto MCNs of SON. (A) The representative traces show the frequency of sIPSCs before and after morphine administration. (B, C) Bath application of morphine (25 μM) elicited a significant reduction in the frequency of sIPSCs but the amplitude of sIPSCs remains unchanged (n=7). The effect of morphine on sIPSC frequency was blocked by bath application of 50 μM naloxone (NAL). (D) Changes in sIPSCs frequency (% of control) during recording. Each bar represents mean ± SEM.

A significantly opposite action of acute bath application and chronic morphine administration was observed on both inhibitory [F(2, 18) = 33.08, p < 0.0001; Figure 1A] and Excitatory [F(2, 15) = 246.1, p < 0.0001; Figure 2B] synaptic activity of MCNs. While acute application of morphine in the bath perfusion suppressed GABAergic synaptic activity in MCNs (p < 0.05) compared to control group, chronic application of morphine increased spontaneous GABAergic activity of these neurons (p < 0.001) compared to control group. For excitatory transmission, acute bath application of morphine significantly increased (p < 0.001), but chronic morphine administration significantly decreased (p < 0.01) synaptic activity compared to control group. 

*Acute application of morphine induced excitation of MCNs*

**Figure 3:**
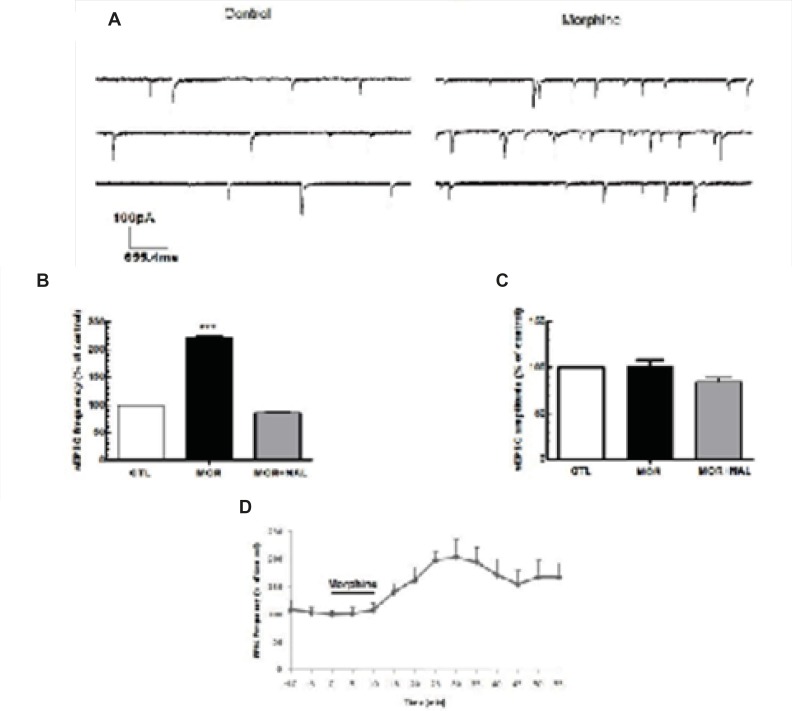
Acute morphine-induced facilitation of glutamate release onto MCNs of SON. (A) Representative traces showing the effect of Morphine on sEPSCs. (B, C) Bath application of morphine (25 μM) elicited an increase in the frequency of sEPSCs but not its amplitude (n=5) and this effect was blocked by naloxone (NAL; 50 μM). (D) Changes in sEPSCs frequency (% of control) during the recording. Each bar represents mean ± SEM.

Acute application of morphine and naloxone produced significant change in frequency [F(2, 18) = 6.673, p = 0.0068; Figure 2B], but no change in the amplitude [F(2, 18) = 0.075, p = 0.927; Figure 2C] of inhibitory currents. Moreover, Acute application of morphine and naloxone produced significant change in frequency [F(2, 15) = 2172, p < 0.0001; Figure 3B], but no change in the amplitude [F(2, 15) = 3.505, p = 0.0564; Figure 2C] of excitatory currents. Bath application of the lowest effective dose of morphine (25 μM) for 10 min caused a significant decrease (32% ± 10.55) in the frequency of sIPSCs (P<0.01, Figure 2A, 2B, 2D) but a significant increase (120% ± 2.18) in the frequency of sEPSCs (P<0.001, Figure 3A, 3B, 3D). Acute bath application of morphine had no effect on amplitudes of either sEPSCs or sIPSCs compared to the control values (Figure 2C, 3C). Moreover, bath application of the opioid receptor antagonist, naloxone (50 μM), alone had no significant effect on sIPSCs and sEPSCs (the results were not shown) but prevented the effect of morphine on both sIPSCs and sEPSCs frequencies (Figure 2B, 3B).

* P<0.05, ***P<0.001 compared to control group.

**p<0.01 compared to control group

***p<0.001 compared to control group.

*Naloxone antagonized the effects of repeated morphine administration*

**Figure 4:**
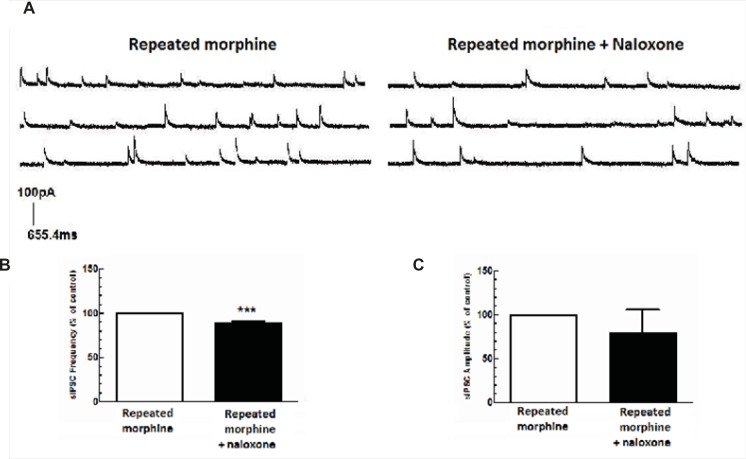
Repeated morphine administration facilitated GABA release in MCNs of SON. The brain slices were obtained from rats treated with repeated doses of morphine. (A) The representative traces show the frequency of sIPSCs before and after naloxone application. (B, C) Average changes in mean sIPSC frequency and amplitude after bath application of naloxone (50 μM) to brain slices of rats treated with repeated morphine administration (n=6). Naloxone significantly changed sIPSC frequency (B), but not sIPSCs aplitude (C) compared to “repeated morphine” group. Each bar represents mean ± SEM.

**Figure 5:**
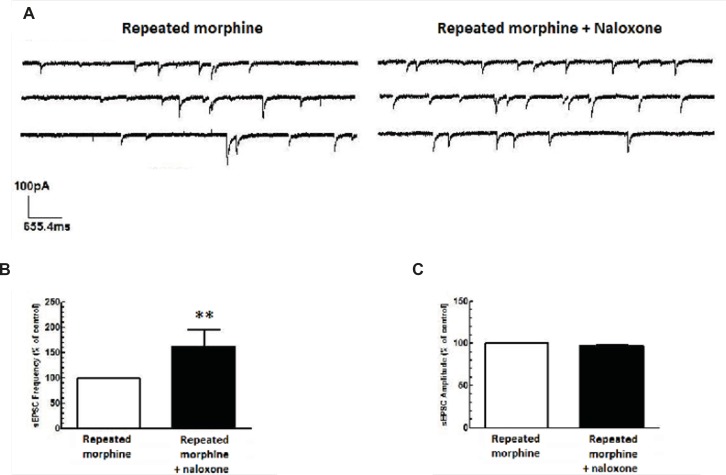
Repeated morphine administration reduced glutamate release in MCNs of SON. (A) The representative traces show the frequency of sEPSCs before and after naloxone application. (B, C) Average changes in sEPSCs frequency and amplitude after bath application of naloxone (50 μM) to brain slices of rats treated with repeated morphine administration (n=5). Naloxone significantly increased sEPSCs frequency (B), but not sEPSCs aplitude (C) compared to repeated morphine group. Each bar represents mean ± SEM.

Repeated administration of morphine increased GABAergic synaptic activity (Figure 1A) but decreased glutamatergic synaptic activity in MCNs (Figure 1B). In brain slices prepared from rats treated with repeated morphine administration, bath application of naloxone (50 μM) to MCNs for 10 min caused a significant decrease in the frequency of sIPSCs [t(10) = 7.765, P<0.001, Figure 4A, 4C) and a significant increase in the frequency of sEPSCs [t(8) = 4.105, P = 0.0034; Figure 5A, 5C] without a significant effect on the amplitude of sIPSCs and sEPSCs (Figure 4B, 5B).

*p<0.001 compared to repeated morphine group

*p<0.01 compared to repeated morphine group

*Effects of i.c.v. morphine administration on plasma AVP levels, urine volume, and water consumption*

**Figure 6:**
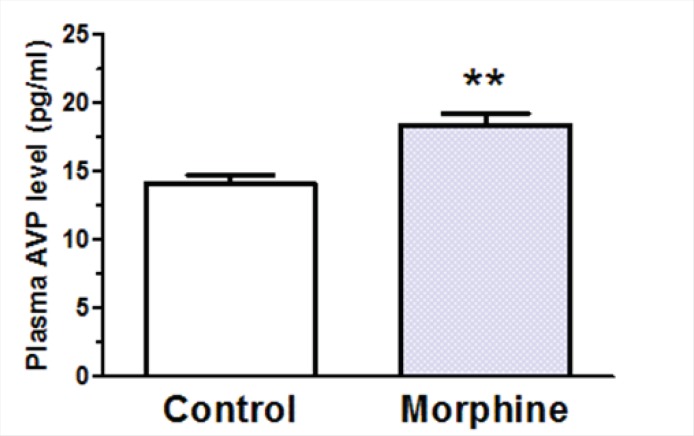
Changes in plasma arginine vasopressin (AVP) levels after repeated i.c.v. administration of morphine for 3 consecutive days. The control group received saline instead of morphine (n=7 in each group). Blood samples were collected 45 min after last morphine administration. Each bar represents mean ± SEM.

**Figure 7:**
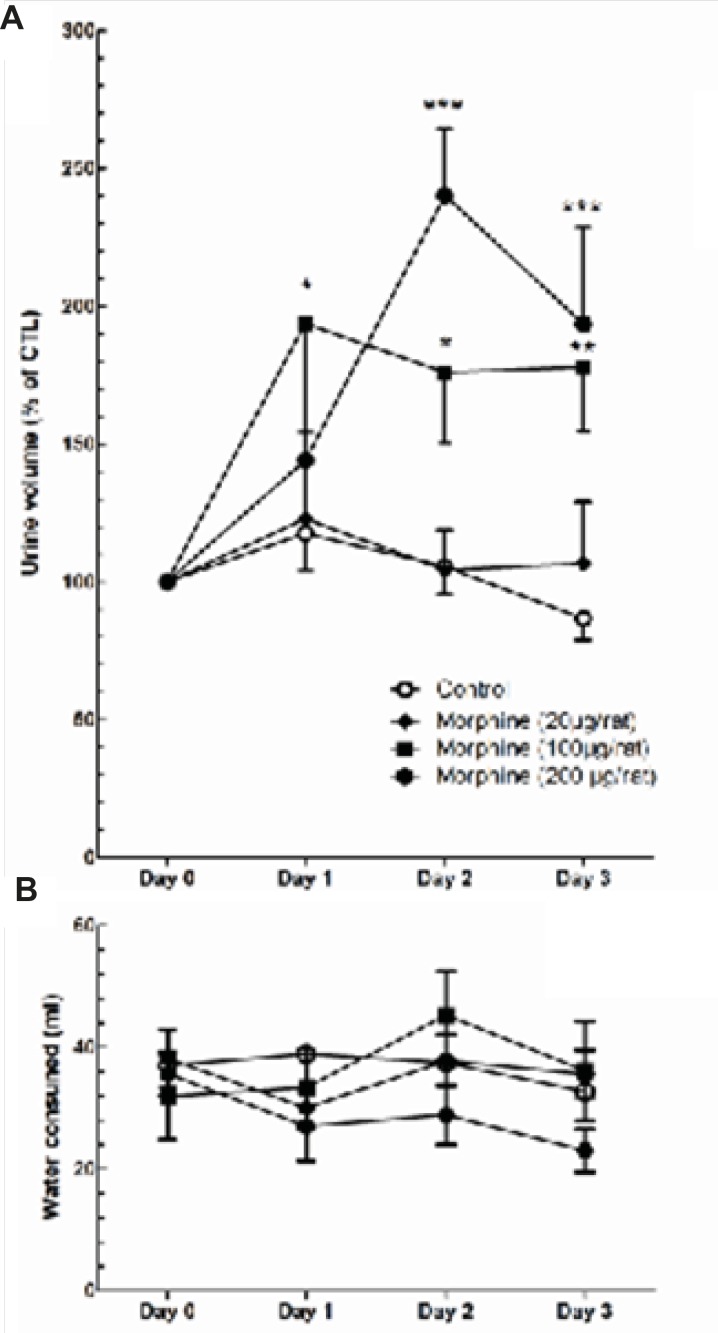
Changes in daily measured urine volume (A) and water consumption (B) during three days of i.c.v. morphine administration. Repeated measure one-way ANOVA followed by Dunnett’s test was used to compare the results of each treatment group with control group. Data are shown as mean ± SEM.

A significant decrease in plasma AVP levels was observed in rats received daily i.c.v. administration of morphine (100 μg/rat) compared to control group which received daily i.c.v. administration of Saline [*t*(12) = 3.928, *p *= 0.002; Figure 6]. Two-way ANOVA revealed that repetitive i.c.v. administration of morphine for three consecutive days caused significant increase in overall volume of daily collected urine [F_treatment_(3, 207) = 15.29, p < 0.0001; Figure 7A]. Further analysis using Bonferroni’s post test revealed significant increase in urine volume in first (p < 0.05), second (p < 0.05) and third (p < 0.01) day in rats received i.c.v. injection of morphine (100 g) compared to control group (Figure 7A). Moreover, significant increase in urine volume was observed in rats treated with i.c.v. administration of morphine (200 μg) in second (p < 0.001) and third (p < 0.001) days of drug injection compared to control group (Figure 7A). Two-way ANOVA revealed no significant alteration in water consumption in morphine treated groups compared to control group [F_treatment_(3,127)=2.085, p=0.105; Figure 6B].

**P<0.01 compared to control group.

## Discussion

In the present study, acute bath application of morphine (25 μM) produced a presynaptic excitatory effect by decreasing the frequency of sIPSCs and by increasing the frequency of sEPSCs without altering their amplitude. On the other hand, *in-vivo *repeated morphine administration for four consecutive days produced a presynaptic inhibitory effect by increasing the frequency of sIPSCs and by decreasing the frequency of sEPSCs without altering their amplitude. Moreover, the effects of morphine on sIPSCs and sEPSCs were inhibited by opioid receptor antagonist, naloxon (50 μM), which indicated an opioid receptor-mediated action. The decrease in MCN activity after repetitive *in-vivo *morphine administration was consistent with a decrease in AVP secretion by these cells followed by an increase in urine volume of rats.

*p < 0.05, **p < 0.01, ***p < 0.001 compared to control group at the same day.

The MCNs in SON are strongly dependent on synaptic inputs to generate their output. Thus, modulation of the synaptic inputs may have great impact on their firing patterns and hormone secretion. It was suggested that opioids effects on MCNs are different depending on the type of cells, opioid receptors and synaptic transmissions (10, 23, 24). The studies have shown that morphine could have either inhibitory or excitatory effects, depending on the protocol of morphine administration (acute or repeated administration). Some reports indicated that acute morphine administration increases expression of AVP, norepinephrine (NE) and c-fos proteins in SON but there is a decreased expression of AVP, NE and c-fos proteins after chronic administration of morphine (13, 19). It was also shown that acute morphine administration (having predominantly mu-agonist activity) induces potent antidiuretic effects in dose-dependent manner (25). However, in some studies acute morphine administration has induced a diuresis effect but it was suggested this effect could be due to an increase of atrial natriuretic peptide (26). On the other hand, the chronic morphine exposure reduces the number of available mu-receptors in the supraoptic nucleus but no significant changes in kappa-selective binding (9). The morphine effects on SON is likely mediated by μ-opioid receptors because κ-opioid receptor agonists produce opposite effects (27, 28) and SON neurons do not express δ-opioid receptors in morphine naïve rats or morphine-dependent rats (9). Furthermore, the selective μ-opioid agonist D-Ala(2), N-CH(3)-Phe(4), Gly(5)-ol-enkephalin (DAGO), decreases the frequency (but not the amplitude) of EPSCs, indicating that glutamatergic inputs to SON neurons are under presynaptic μ-opioid inhibition (23). According to a recent study using sharp electrode recording, SON neurons were shown to be inhibited when chronically exposed to morphine, and undergo withdrawal excitation when morphine is subsequently acutely antagonized by naloxone (12). On the other hand, acute exposure to morphine (5 μM) increased after depolarization amplitude in SON neurons by about 80% (12).

Different effects of morphine on excitatory or inhibitory presynaptic terminals could be related to specificity of type of opioid receptors expressed on terminals or activation of different signaling pathways. As mentioned earlier, morphine predominantly affects the mu-receptors and it has been shown that the chronic administration of morphine decreases the number of available mu-receptors (9). Although a decrease in the number and affinity of mu-receptors was observed during repeated morphine administration in such brain regions as striatum (29), this event was not demonstrated in SON and needs further experiments to be clarified. Another suggested mechanism is that mu-opioid receptors activate α subunits in different types of G-proteins and signaling pathways (30, 31); thus the different effects of morphine on excitatory and inhibitory presynaptic terminals at the SON might be due to activation of different signaling pathways induced by mu receptors.

Previous studies showed chronic exposure to morphine could develop dependence in oxytocin-releasing MCNs of SON (32, 33). In contrast to oxytocin neurons, there is no sufficient evidence regarding dependency of SON vasopressin neurons after chronic morphine treatment (34, 35), yet some studies suggest a modest increase in plasma vasopressin concentrations after repeated morphine administration (34).

Our results regarding morphine-induced increase in the amount of urine volume was consistent with the changes in the activities of MCNs after repeated exposure to morphine. A decrease in the activity of MCNs after repeated morphine administration resulted in a decrease in plasma AVP levels and eventually an increase in urine volume. No significant change in daily water consumption was observed which suggests MCNs activity and plasma AVP are not the sole factors determining the amount of water consumption in rats.

This study showed complex effects of morphine on SON neurons. While acute morphine increased the activity of SON neurons, repeated morphine administration produced opposite effect. Also, induction of withdrawal from repeated morphine administration (by addition of naloxone to bath perfusion) produced neuronal excitation in SON presented as increased EPSCs and decreased IPSCs. Acute morphine-induced excitation was consistent with the results of previous studies (10, 12). Also, the naloxone-induced withdrawal of repeated morphine was consistent with the observed changes in post-spike excitability (35) as well as increase in after depolarization amplitude (12) seen in SON neurons. Taken together with the plasma AVP level results, our current results suggest that decreased plasma AVP levels after repeated morphine exposure could be due to its suppressor effect on MCNs. A morphine-induced gradual decrease in synaptic excitatory but increase in synaptic inhibitory inputs may lead to diminish in the activation of MCNs and this effects could be withdrawn with naloxone. These results might explain in part the central mechanism by which morphine could increase urine volume in rats.
